# Protective Effects of Aminooxyacetic Acid on Colitis Induced in Mice with Dextran Sulfate Sodium

**DOI:** 10.1155/2021/1477345

**Published:** 2021-12-10

**Authors:** Wenyang Li, Jianghong Yu, Bohan Jin, Huilu Zhang, Jun Zhang

**Affiliations:** Department of Digestive Diseases, Huashan Hospital, Fudan University, Shanghai, China

## Abstract

As a known inhibitor of pyridoxal phosphate-dependent transaminase glutamic-oxaloacetic transaminase 1 (GOT1), aminooxyacetic acid (AOAA) has been pointed out to have potential pharmacological effects in antiepileptic, anticonvulsant, antibacterial, cancer cell proliferation inhibition, and acute myocardial infarction (MI) relief. However, its role in inflammatory bowel disease (IBD) has not been reported. Through the in vivo experiment of dextran sulfate sodium- (DSS-) induced colitis in mice, it was found that AOAA significantly attenuated the symptoms, signs, and pathological changes of colitis. In addition, AOAA treatment prevented gut barrier damages by enhancing the expression of zona occludens- (ZO-) 1, occludin, claudin-1, and E-cadherin and recovering the upregulation of the most abundant intermediate filament protein (vimentin). Moreover, the release of interleukin- (IL-) 1*β*, IL-6, and tumour necrosis factor- (TNF-) *α* was suppressed, yet the level of IL-10 was upregulated by AOAA treatment compared to the model group. Furthermore, it was shown that AOAA administration boosted M2-like phenotype and effectively reduced M1 macrophage phenotype in the lamina propria of mouse colonic epithelium. Similarly, the effect of AOAA was verified in vitro. AOAA effectively inhibited the classically activated M1 macrophage phenotype and proinflammatory cytokine (IL-1*β*, TNF-*α*, and IL-6) expression induced by lipopolysaccharide (LPS) and promoted M2-like phenotype. Collectively, this study reveals for the first time that short-term treatment of AOAA can significantly alleviate DSS-induced acute colitis by regulating intestinal barrier function and macrophage polarization, which provides a theoretical basis for the potential use of AOAA in the treatment of IBD.

## 1. Introduction

Inflammatory bowel disease (IBD) is further divided into Crohn's disease (CD) and ulcerative colitis (UC) and is a chronic disease responsible for inflammation of intestinal tract [1]. It can occur in people of any age from infancy to 80s and seriously affects the health and life quality of human beings [2]. At present, about 1.6 million residents are affected with IBD in the USA, while in Europe, as many as 2 million people suffer from the disease [3]. In developing countries, the prevalence of IBD is still lower, but the incidence rate is higher and higher due to expanding urbanization and westernization, which indicates that the number of IBD cases in developing countries may exceed that in developed countries one day in the future [4]. Therefore, IBD is becoming a worldwide health threat with rising incidence and bringing huge burden and costs to the health-care system [3].

Treatment strategies for IBD focus on the induction and long-term maintenance of deep remission to avoid complications of active disease and improve long-term outcomes [5]. 5-Amino salicylate, corticosteroids, immunosuppressive agents and antitumour necrosis factor (TNF), antibiotics, antioxidants, probiotics, phosphodiesterase inhibitors, potassium channel openers, adenosine triphosphate donors, melatonin, and some natural products are some of the current available therapeutic options for IBD [5]. However, these therapies are involved with more or less insufficient efficacy or have somehow safety concerns, which together with other factors—including increasing treatment costs and patient preferences—leads to concerns regarding indefinite use of medical therapy [6]. Therefore, except for nonresponders, discontinuation of treatment due to lack of drug potential to maintain patients in remission in long term is usually observed in clinical practice [7]. Besides, as addressed above, some toxic adverse events and sensitivity reactions make conventional therapeutics intolerable for patients with chronic active CD or UC. To sum up, it is urgent to develop new therapeutic strategies.

The gastrointestinal barrier is crucial to prevent the entry of pathogenic microorganisms and toxic luminal substances, while allowing efficient transport of nutrients across the epithelium [8]. When breached, microorganisms and toxins within the lumen invade the lamina propria or even the systemic circulation and result in cytokine stress or inflammation of the intestinal mucosa [9]. It has been reported that intestinal epithelial permeability increased in mice with IBD and which has often been related to changes in tight junction (TJ) proteins within the epithelium, such as zona occludens- (ZO-) 1, occludin, and claudin-1 [10]. Besides, a previous study has suggested that there is a direct relationship between intestinal epithelial-to-mesenchymal transition (EMT) and the pathogenesis of IBD, because EMT marker E-cadherin is downregulated, whereas vimentin is upregulated during the loss of cell adhesion [11]. In addition, the gastrointestinal tract also is home to the largest compartment of the immune system [12]. Under normal circumstances, the balance of the immune system can prevent the occurrence of diseases, such as the balance of proinflammatory cytokines and anti-inflammatory cytokines in regulating inflammation [13]. As a key outpost of intestinal immunity, macrophages play an important role in both innate and adaptive immune responses. Macrophages can be activated to proinflammatory M1 phenotype or anti-inflammatory M2 phenotype, by lipopolysaccharide (LPS) or interleukin- (IL-) 4/IL-10, respectively [14, 15]. These two phenotypes switch between each other during the inflammatory response, and the imbalance of the transformation of M1/M2 macrophages is increasingly regarded as the main factor leading to IBD [12]. Thus, regulating intestinal barrier function and the balance between M1 and M2 macrophages is considered as a potential strategy for the treatment of IBD.

Aminooxyacetic acid (AOAA) inhibits pyridoxal-5′-phosphate-dependent transaminases, which mediate the interconversion of *α*-amino and *α*-keto acids in a reductive amination, in which the redox balance of the reaction is maintained by concomitant conversion of glutamate (nitrogen donor) into *α*-ketoglutaric acid (*α*-KG) [16]. It has been pointed out to have a variety of pharmacological effects. For example, it can significantly attenuate experimental autoimmune encephalomyelitis (EAE) by regulating the fate of T cells [16]. Interestingly, it has been shown that AOAA can reduce cardiac dysfunction after myocardial infarction by regulating macrophage metabolism and balancing macrophage polarization in mice [17], suggesting that the role of AOAA in the immune environment cannot be ignored. However, the effect of AOAA on IBD is still unclear. Based on these theories mentioned above, we tried to explore the effects as well as the mechanisms of AOAA in sodium dextran sulfate- (DSS-) induced acute colitis mice and LPS-stimulated RAW264.7 macrophages/peritoneal macrophages in this study.

## 2. Materials and Methods

### 2.1. Animal Model

Male C57BL/6 mice (6-8 weeks, 20 ± 1 g) were obtained from GemPharmatech Co., Ltd. (Nanjing, China). All mice were caged under specific pathogen-free conditions and were conducted in accordance with the guidelines on the care and use of laboratory animals. The procedures of all animal experiments in this experiment were approved by the Animal Ethics Committee of Fudan University (2018, Huashan Hospital, JS-092). In brief, mice were randomly divided into 4 groups (H_2_O+PBS, H_2_O+5 mg/kg AOAA, DSS+PBS, and DSS+5 mg/kg AOAA) with 5 mice in each group. For inducing acute colitis, mice were fed with 3% (w/v) DSS (CAT No. 160110, MP Biomedicals, USA) in their drinking water for 7 days and carefully monitored daily to confirm that their intake of DSS-containing water was approximately the same volume, as previously stated [18, 19]. The third day after mice were induced by DSS or not, AOAA (C13408, Sigma-Aldrich, USA) was dissolved in PBS and injected intraperitoneally (5 mg/kg) for 5 days. On the 7th day, the mice were sacrificed by cervical dislocation and then specimens of colon and spleen were collected. In the course of the experiment, the body weight, stool characteristics, and blood in feces and anus of the mice were observed every day from day 1 to day 7. The disease activity index (DAI) is calculated as shown in supplementary Table 1 [20].

### 2.2. Cell Culture and Treatment

RAW264.7 macrophages (Xiangya Central Laboratory Cell Bank, Central South University, Changsha, China) were cultured in DMEM medium (37°C) containing 10% fetal bovine serum (10099141C, Gibco, USA), 100 U/mL penicillin, and 100 *μ*g/mL streptomycin (SV30010, HyClone, USA) [21]. Peritoneal macrophages were obtained from mice by intraperitoneal injection of 2 mL mercaptoacetate broth for 3 days and were isolated as described [20]. Cells were pretreated with AOAA (1, 5 mM) for 1 h before LPS (1 *μ*g/mL) stimulation, and samples were collected 24 h later.

### 2.3. Cytokine Analysis by ELISA

After being treated with AOAA, whole blood samples were collected from the medial canthus vein. Immediately, blood samples were centrifuged at 12,000 × g for 10 min at 4°C, and serum samples were aspirated and aliquoted and then stored at −80°C. Similarly, the supernatants of cultured cells were collected and stored at −80°C. Levels of IL-1*β*, IL-6, tumour necrosis factor- (TNF-) *α*, and IL-10 were measured using the ELISA kits (Hangzhou Lianke Biotechnology Co., Ltd.), as described previously [18].

### 2.4. Histopathology

All colon tissues were taken from a distal colonic site close to the rectum (2 mm × 6 mm) and fixed in 4% paraformaldehyde. Samples were embedded in paraffin and sectioned (4 *μ*m) then stained with hematoxylin and eosin (HE) [22, 23]. The histological changes were observed with optical microscopy (Olympus, Tokyo, Japan). The criteria were used to assess colitis using a standard histological scoring system. Briefly, for infiltration of inflammatory cells, rare inflammatory cells in the lamina propria were counted as 0, increased numbers of inflammatory cells in the lamina propria were counted as 1; confluence of inflammatory cells extending into the submucosa was counted as 2; a score of 3 was given for transmural extension of the infiltrate. For tissue damage, no mucosal damage was counted as 0, discrete lymphoepithelial lesions were counted as 1, surface mucosal erosion was counted as 2, and a score of 3 was given for extensive mucosal damage and extension through deeper structures of the bowel wall. The combined histologic score ranged from 0 (no changes) to 6 (extensive cell infiltration and tissue damage) [24].

### 2.5. RNA Preparation and qRT-PCR

The total RNA in colon tissue was extracted using Trizol (Invitrogen, USA), and RNA was reverse-transcribed into cDNA using RNA reverse transcription kits, according to previous research [21]. The mRNA levels of IL-1*β*, IL-6, TNF-*α*, IL-6, IL-10, CD80, CD206, Arg1, iNOS, ZO-1, occludin, claudin-1, E-cadherin, and vimentin genes were examined on a Bio-Rad Q5 instrument (Bio-Rad, CA, USA) using a SYBR Premix EX Taq Realtime PCR Master Mix (TaKaRa). The primer sequences are described in Supplemental Table 2. The 2-*ΔΔ*Ct formula was used to normalize target gene transcription to *β*-actin expression (internal control) to calculate fold change of target mRNA.

### 2.6. Immunohistochemical Staining

The sections of colon tissue were blocked by 3% H_2_O_2_ and goat serum; then, the samples were incubated with the following primary antibodies at 4°C overnight: ZO-1 (1 : 200, GB111981, Servicebio), occludin (1 : 600, GB111401, Servicebio), claudin-1 (1 : 400, GB11032, Servicebio), E-cadherin (1 : 500, GB12082, Servicebio), and vimentin (1 : 1000, GB12192, Servicebio). Then, sections were washed with PBS and incubated with the appropriate secondary antibody (Dako Real Envision/HRP, Rabbit/Mouse, K5007) at room temperature for 1 h. After that, 3,3′-diaminobenzidine with peroxidase substrate (Dako Real DAB Chromogen, K5007) were used for chromogenic reaction. Counter staining was performed with hematoxylin.

### 2.7. Isolation of Lamina Propria Mononuclear Cells (LPMCs)

LPMCs were separated using the method described previously [25]. In short, the colons were removed from the executed mice, cut into 0.5 cm pieces, and thoroughly washed with cold PBS to remove all feces and blood [26]. Then, they were incubated with 2 mM DTT and 1 mM EDTA in 37°C PBS for 2 × 20 min with gently shaken to remove intestinal epithelial cells and digested with 10 mL 2% fetal bovine serum-RPMI-collagenase A (1 mg/mL, Roche, Mannheim, Germany) in a 37°C incubator for 30 minutes [27]. The lamina propria cells were then collected and further purified by density gradient centrifugation with 40% Percoll-RPMI solution [27]. LPMCs were collected from the middle layer and stored in dry ice until used.

### 2.8. Flow Cytometry

The following fluorescent-labeled monoclonal antibodies and staining reagents are used according to the manufacturer's specifications: anti-APC-CY7-live (BioLegend), anti-BV510-CD45 (BioLegend), anti-F4/80-BV421 (BioLegend), anti-CD11B-PE CY7 (BioLegend), anti-CD206-APC (BioLegend), anti-CD80-percp/Cy5.5 (BioLegend), anti-LY6C-APC (BioLegend), anti-CD11B-PE (BioLegend), anti-LY6G-PE CY7 (BioLegend), anti-CD3-PE CY7 (BioLegend), anti-CD4-BV421 (BioLegend), anti-CD8-percp/CY5.5 (BioLegend). The cells were proceeded by FACS Calibur flow cytometry and analyzed by the FlowJo software.

### 2.9. Statistical Analysis

GraphPad Prism 8.0 and SPSS 22.0 were used to analyze dates. Results were presented as the mean ± standard derivation for at least three separate experiments. One-way analysis of variance (ANOVA) was used to determine statistically significant differences. *P* < 0.05 was considered significant.

## 3. Results

### 3.1. AOAA Alleviates Symptoms and Signs in DSS-Induced Mouse Colitis Model

**Figure 1:**
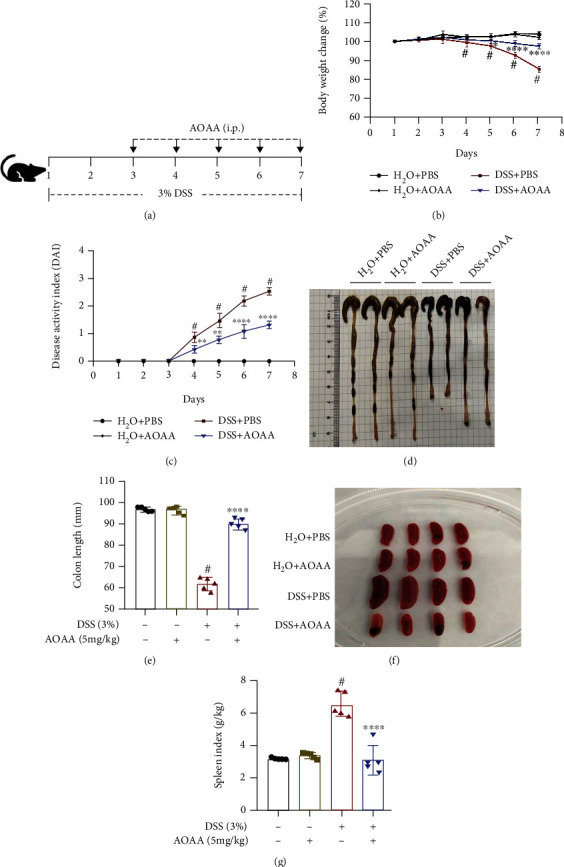
AOAA attenuates signs and symptoms in DSS-induced colitis model. (a) Schematic overview of the acute colitis regimen. (b) Loss of basal body weight after DSS induction. (c) DAI evaluations. (d) Macroscopic appearances of colons. (e) Colon length. (f) Macroscopic appearances of spleens. (g) Spleen weight index. One representative experiment of three is shown. Data are presented as mean ± SD (*n* = 5) (^#^*P* < 0.05 vs. the H_2_O+PBS group; ^∗^*P* < 0.05, ^∗∗^*P* < 0.01, and ^∗∗∗∗^*P* < 0.0001 vs. the DSS+PBS group).

To determine the role of AOAA in IBD, we used the 3% DSS-induced mice acute colitis model (Figure 1(a)) and monitored body weight, DAI, colon length, and spleen index for 7 days. As expected, there was no difference in the H_2_O+AOAA group compared with the H_2_O+PBS group, whereas the signs and symptoms of mice in the DSS model group were significantly worse than those in the H_2_O+PBS group. These mice exhibited a reduction in daily activity, anorexia, drab hair color, and weight loss with a loose stool. However, compared with the DSS+PBS group, it was shown that weight loss (Figure 1(b)), DAI increase (Figure 1(c)), colon shortening (Figures 1(d) and 1(e)), and splenomegaly (Figures 1(f) and 1(g)) were significantly alleviated in the DSS+AOAA group.

### 3.2. AOAA Alleviated Histopathological Changes and Restored Gut Barrier Integrity in DSS-Induced Colitis Mice

**Figure 2:**
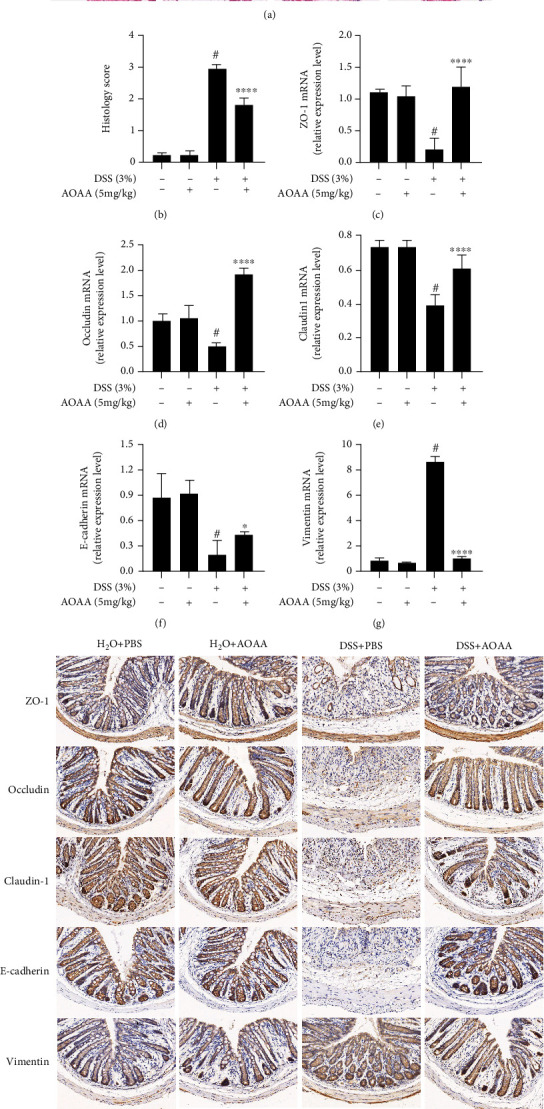
AOAA alleviated DSS-induced colitis in mice and restored mRNA expression of epithelial barrier. (a) Colonic sections were stained with H&E (original magnification ×200). (b) Histopathological scores of colonic sections. The mRNA expression of (c) ZO-1, (d) occludin, (e) claudin-1, (f) E-cadherin, and (g) vimentin. (h) The expressions of these proteins were tested by immunohistochemistry in experimental colitis in mice. One representative experiment of three is shown. Data are represented as mean ± SD (*n* = 5) (^#^*P* < 0.05 vs. the H_2_O+PBS group; ^∗^*P* < 0.05, ^∗∗^*P* < 0.01, and ^∗∗∗∗^*P* < 0.0001 vs. the DSS+PBS group).

In order to further study the inhibitory effect of AOAA on colonic inflammation and ulcer, colonic histology was evaluated. It was shown that there was no difference between the H_2_O+PBS group and the H_2_O+AOAA group, but DSS-treated mice exhibit epithelial cell destruction, crypt deformation, ulcer formation, and inflammatory cell infiltration (mainly mononuclear macrophages, neutrophils, and eosinophils) in lamina propria and submucosa compared with the H_2_O+PBS group. However, after treatment with AOAA, the degree of colonic mucosal lesion was significantly alleviated, the infiltration of inflammatory cells in mucosa and submucosa was significantly reduced, and the integrity of colonic mucosa was maintained (Figures 2(a) and 2(b)).

A previous study proved that the damage of gut barrier integrity was the prime pathological characteristic of colitis, which resulted in the exposure of epithelium to bacteria and toxins from lumen [8]. To determine the effects of AOAA on gut barrier integrity in DSS-induced colitis mice, the expression of ZO-1, occludin, claudin-1, E-cadherin, and vimentin was detected by RT-PCR and immunohistochemistry, respectively. Compared with the H_2_O+PBS group, the downregulation of ZO-1, occludin, claudin-1, and E-cadherin and upregulation of vimentin mRNA levels were observed in the DSS+PBS group, while the administration of AOAA significantly restored the mRNA levels of these genes (Figures 2(c)–2(g)). Consistently, the results showed that administration of AOAA significantly increased the protein expression levels of ZO-1, occludin, claudin-1, and E-cadherin and decreased the protein expression of vimentin compared to the model group by immunohistochemistry staining (Figure 2(h)). It should be noted that the above results did not differ between the H_2_O+PBS group and the H_2_O+AOAA group.

### 3.3. AOAA Inhibits DSS-Induced Colonic Inflammation

**Figure 3:**
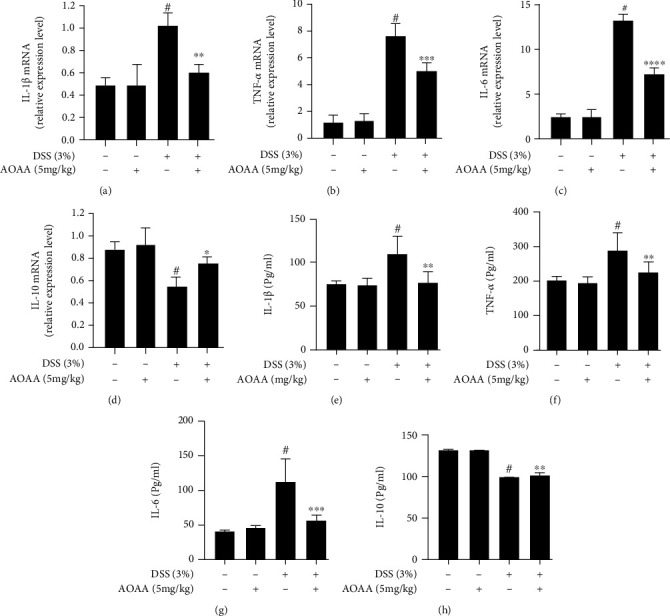
AOAA inhibits inflammation in DSS-induced colitis. The mRNA expression of cytokines (a) IL-1*β*, (b) TNF-*α*, (c) IL-6, and (d) IL-10 in colonic tissues. Protein levels of cytokines including (e) IL-1*β*, (f) TNF-*α*, (g) IL-6, and (h) IL-10 in colonic homogenates. One representative experiment of three is shown. Data are presented as mean ± SD (*n* = 5) (^#^*P* < 0.05 vs. the H_2_O+PBS group; ^∗^*P* < 0.05, ^∗∗^*P* < 0.01, ^∗∗∗^*P* < 0.001, and ^∗∗∗∗^*P* < 0.0001 vs. the DSS+PBS group).

To identify whether the outcome of colitis in mice treated with AOAA is related to the reduction of inflammatory factors associated with colitis, the transcription and expression of IBD-related inflammatory cytokines in colon tissue were detected by qPCR and ELISA, respectively. PCR results showed there was no difference between the H_2_O+PBS group and the H_2_O+AOAA group, and higher IL-1*β*, TNF-*α*, and IL-6 levels were found in the DSS+PBS group compared with the H_2_O+PBS group, but they were dramatically decreased by treatment of AOAA (Figures 3(a)–3(c)). However, DSS significantly decreased the mRNA expression of IL-10 compared with the H_2_O+PBS group (Figure 3(d)). The expression of IL-10 mRNA in the DSS+AOAA group increased approximately 41% compared with the model group (Figure 3(d)) (*P* < 0.05). Similarly, ELISA results suggested that IL-1*β*, TNF-*α*, and IL-6 levels were noteworthy higher in the DSS+AOAA group than in the H_2_O+PBS group but were lower than in the DSS+PBS group (Figures 3(e)–3(g)). After the treatment with AOAA, the protein expression of IL-10 reached 102.95 ± 1.64, which was remarkably higher than that in the DSS+PBS group (99.67 ± 0.25, *P* < 0.01) (Figure 3(h)). Collectively, these results indicated that AOAA plays a role in balancing proinflammatory cytokines and anti-inflammatory cytokines.

### 3.4. AOAA Restored the Changes of Intestinal Local Immune Pattern in DSS-Induced Colitis Mice

**Figure 4:**
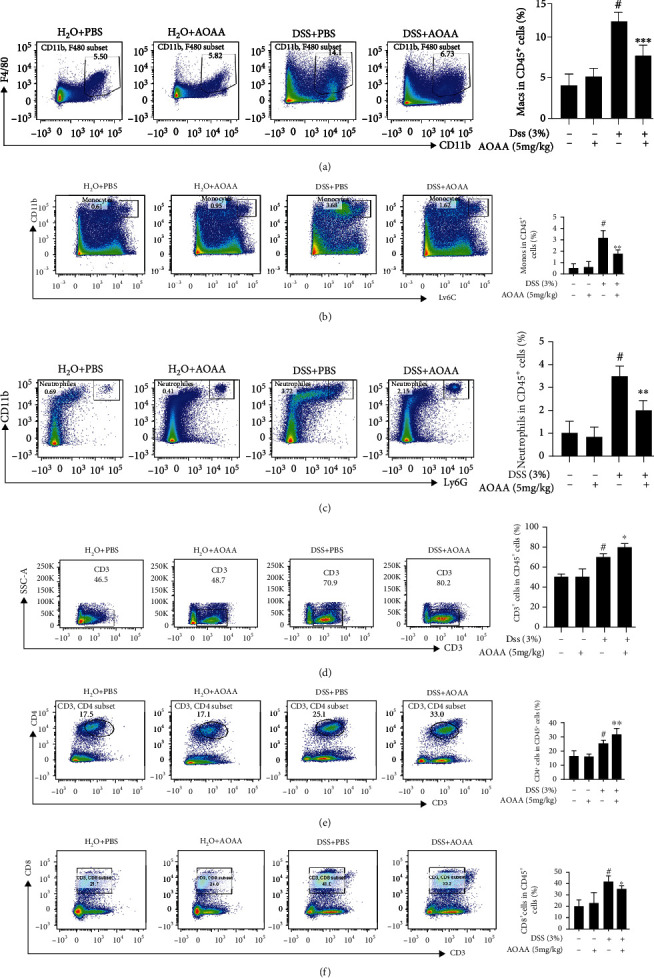
AOAA restored the changes of intestinal local immune pattern of DSS-induced colitis. The percentage of (a) macrophages (CD11b^+^ and F4/80^+^ cells), (b) monocytes (CD11b^+^ and Ly6c^+^ cells), and (c) neutrophils (CD11b^+^ and Ly6G^+^ cells), (d) T cells (CD3^+^ cells), (e) CD4^+^ T cells (CD3^+^ and CD4^+^ cells), and (f) CD8^+^ T cells (CD3^+^and CD8^+^ cells) in colonic LP were examined by FACS analysis. One representative experiment of three is shown. Data in (a–f) (right panels) are shown as mean ± SD (*n* = 5) (^#^*P* < 0.05 vs. the H_2_O+PBS group; ^∗^*P* < 0.05, ^∗∗^*P* < 0.01, and ^∗∗∗^*P* < 0.001 vs. the DSS+PBS group).

Immune cells mainly exist in the lamina propria of intestinal mucosa, which represent the inflammatory site of IBD [3]. Therefore, flow cytometry was used to detect the changes of immune cells in the lamina propria of colonic mucosa of mice. It was shown that there was no difference between the H_2_O+PBS group and the H_2_O+AOAA group, and the percentages of macrophages, monocytes, neutrophils, and T cells in DSS-induced colitis mice were significantly higher than those in the H_2_O+PBS group (Figures 4(a)–4(f)). Interestingly, as shown in Figure 4, although AOAA treatment further increased the percentage of T cells and CD4^+^ T cells in colitis mice compared with the DSS+PBS group (Figures 4(d) and 4(e)), it declined 47% of macrophages (*P* < 0.001) (Figure 4(a)), 41% of monocytes (*P* < 0.01) (Figure 4(b)), 26% of neutrophils (*P* < 0.01) (Figure 4(c)), and 20% of CD8^+^ T cells (*P* < 0.05) (Figure 4(f)), respectively, suggesting that the local immune environment of the colon was improved and the difference of macrophages was the most significant.

### 3.5. AOAA Regulates the Polarization of Macrophages

**Figure 5:**
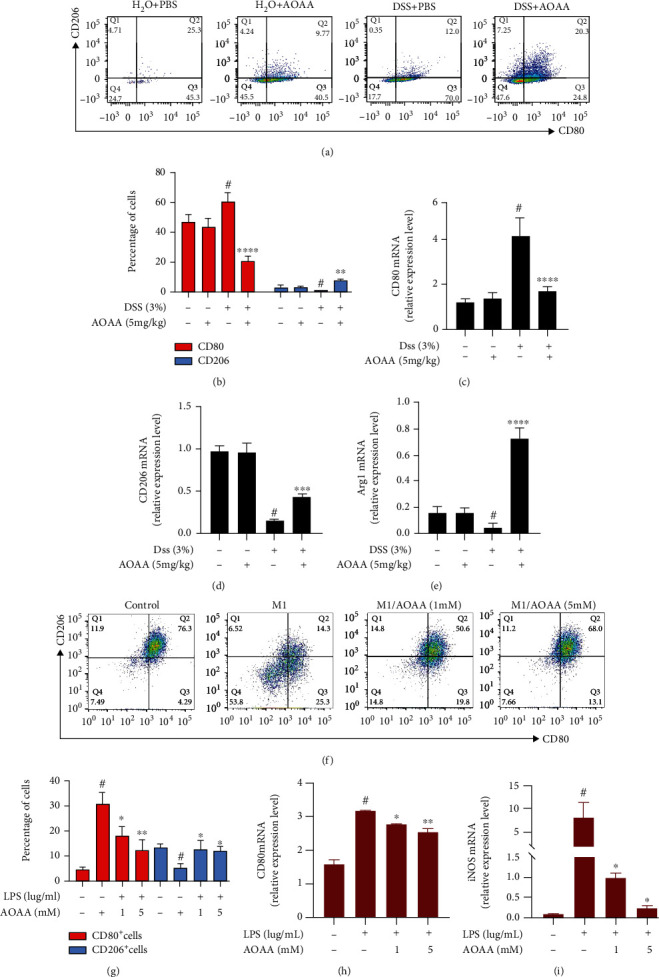
AOAA reprograms M1 macrophage differentiation towards M2 macrophages. (a) AOAA reduced M1 macrophage phenotype and reciprocally increased M2 macrophage phenotype in vivo. (b) Statistics of cell populations from (a). The mRNA expression of (c) CD80, (d) CD206, and (e) Arg1. (f) AOAA reversed the effect of LPS on M1 macrophage differentiation. (g) Statistics of cell populations from (f). The mRNA expression of (h) CD80 and (i) iNOS from cells in (f). In (a–e), data are presented as mean ± SD (*n* = 5) and one representative experiment of three is shown (^#^*P* < 0.05 vs. the H_2_O+PBS group; ^∗∗^*P* < 0.01 and ^∗∗∗∗^*P* < 0.0001 vs. the DSS+PBS group.) In (f–i), data are presented as mean ± SD of three independent repeats (^#^*P* < 0.05 vs. the untreated cell group; ^∗^*P* < 0.05 and ^∗∗^*P* < 0.01 vs. the LPS-stimulated group).

After knowing the effects of AOAA on macrophage, we analyzed the percentages of CD80-positive (M1 macrophage marker) and CD206-positive (M2 macrophage marker) cells in the lamina propria of mouse colon by flow cytometry to further verify the possible protective mechanism of AOAA on DSS-induced acute colitis. It was shown there was no difference between the H_2_O+PBS group and the H_2_O+AOAA group, and CD80-positive cells reached to 22.32 ± 1.81% in the DSS+AOAA group, which was remarkable lower than that of the DSS+PBS group (53.52 ± 0.99%, *P* < 0.0001), while CD206-positive cells (2.99 ± 0.14%) in the DSS+AOAA group increased significantly compared with the DSS+PBS group (0.4 ± 0.03%, *P* < 0.01) (Figures 5(a) and 5(b)). Also, iNOS is a characteristic marker of M1 phenotype, and Arg1 is a well-reported and established marker of alternative activated M2 macrophages. Expression of CD80, CD206, and Arg1 was determined by qPCR, which showed consistent results (Figures 5(c)–5(e)).

Moreover, the above results were also confirmed in vitro. Cells were pretreated with AOAA (1, 5 mM) for 1 h before LPS (1 *μ*g/mL) stimulation for 24 hours, as described obviously [28]. FACS analysis indicated that after the stimulation with LPS, CD80 and CD206-positive cells changed from 4.78 ± 0.01% and 13.37 ± 0.34% to 30.37 ± 0.25% and 5.65 ± 0.34% (all *P* < 0.05) (Figures 5(f) and 5(g)). However, AOAA pretreatment evidently decreased the percentage of CD80-positive cells and increased the percentage of CD206-positive cells in classic activated M1 macrophages (Figures 5(f) and 5(g)). At last, we evaluated the expressions of M1-associated anti-inflammatory cytokines by qPCR. We found that the expressions of CD80 and iNOS were dramatically reduced by AOAA (Figures 5(h) and 5(i)). These findings suggest that AOAA plays a role in alleviating DSS-induced colitis by restraining M1 macrophage phenotype and boosting M2-like phenotype to adjust macrophage polarization.

### 3.6. AOAA Inhibits LPS-Induced Transcription and Expression of Proinflammatory Cytokines in RAW264.7 Macrophages and Peritoneal Macrophages

**Figure 6:**
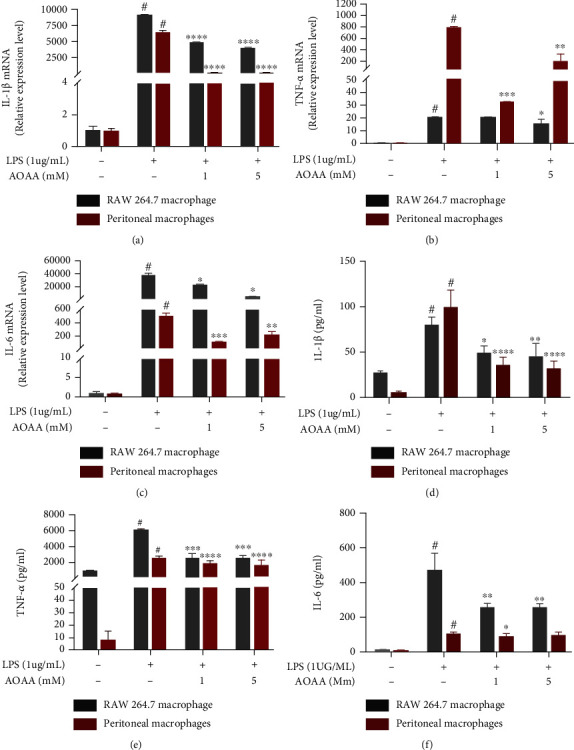
AOAA inhibits the transcription and translation of proinflammatory cytokines in LPS-induced RAW 264.7 macrophages and peritoneal macrophages. The mRNA expression of cytokines (a) IL-1*β*, (b) TNF-*α*, and (c) IL-6 in cells. Protein levels of cytokines including (d) IL-1*β*, (e) TNF-*α*, and (f) IL-6 in cell culture supernatant. Data are presented as mean ± SD of three independent repeats (^#^*P* < 0.05 vs. the untreated cell group; ^∗^*P* < 0.05, ^∗∗^*P* < 0.01, ^∗∗∗^*P* < 0.001, and ^∗∗∗∗^*P* < 0.0001 vs. the LPS-stimulated group).

We examined the effects of AOAA on the production and mRNA expression of IL-1*β*, TNF-*α*, and IL-6 in LPS-induced macrophages. After RAW264.7 macrophages or peritoneal macrophages were stimulated by LPS for 24 hours, it was found that the transcription and expression of proinflammatory cytokines (IL-1*β*, TNF-*α*, and IL-6) were significantly upregulated. However, by pretreatment with AOAA, the transcription and expression of these proinflammatory cytokines were significantly restored (Figures 6(a)–6(f)). These results suggest that AOAA may exert immunosuppressive effect in vitro by inhibiting the release of proinflammatory cytokines from LPS-induced macrophages, which is consistent with the findings in vivo.

## 4. Discussion

AOAA first appeared as a potentially effective pharmacological tool for the inhibition of pyridoxal phosphate- (PLP-) dependent enzymes [29]. It has been confirmed that AOAA reduced the production of lactic acid, inhibited the production of pentose phosphate pathway (PPP), and increased the production of ATP [29]. Interestingly, M1 macrophages are characterized by enhanced aerobic glycolysis (conversion of glucose to lactic acid) and increased PPP flux, while M2 macrophages have lower glycolysis and PPP than M1 macrophages [30]. Recently, some researchers have proposed that bilobalide, a unique Ginkgo biloba constituent, improved experimental colitis via inhibition of M1 macrophage polarization through the NF-*κ*B signaling pathway [31]. These results suggest that AOAA may balance macrophage polarization through modulating macrophage metabolism and inhibiting NF-*κ*B signal pathway. In this study, in DSS-induced colitis mice treated with AOAA, M1 macrophages in colonic lamina propria were significantly suppressed, while M2 macrophages were increased. We confirmed and extended previous reports about the modulating role of AOAA on macrophage polarization and the role of macrophages in IBD.

A large number of reports have pointed out that the EMT plays an important role in IBD-related intestinal fibrosis and intestinal fistula formation [32]. The process of EMT is characterized by the acquisition of a fibroblast-like elongated morphology, loss of cell polarity, enhanced migratory and invasive capacity, downregulation in the expression of epithelial markers such as adherens and TJ, and upregulation of marker genes of mesenchymal cells [8]. In this study, the expression of the four main TJ proteins (ZO-1, occludin, claudin-1, and E-cadherin) is found to be improved by AOAA. In addition, AOAA also restored the expression of vimentin in mouse colonic tissue with DSS-induced colitis. These results suggest that AOAA not only inhibits the infiltration of inflammatory cells in DSS-induced colitis but also improves intestinal barrier function, paralleled with the modification of macrophage polarization.

AOAA was enrolled in this study due to its strong inhibitory effect on GOT1. Studies have shown that GOT1 as one of the key enzymes in malate shuttle in humans uses PLP as a cofactor to catalyze the conversion of aspartic acid and *α*-ketoglutaric acid to oxaloacetic acid and glutamic acid [33]. By targeting GOT1, AOAA provides a way to inhibit a variety of diseases, which have been widely studied in vitro and in vivo [29]. However, there are many concerns about its inhibition specificity as it inhibits many other enzymes, most of which are achieved through the reaction with PLP at the active center. Thus, the role of AOAA is unlikely to be attributed to any single enzyme or pathway [17]. For example, a recent study showed that administration of AOAA for three consecutive days improved cardiac function in patients with myocardial infarction, in which the cardioprotection effect of AOAA was attributed to its inhibition of malic acid-aspartate shuttle [17]. Therefore, it is warranted to further investigate the protective mechanisms of AOAA against colitis using specific inhibitors of different enzymes separately. In summary, the role of AOAA in IBD is complex and needs further elucidation in the future.

## 5. Conclusion

Our study demonstrated for the first time that short-term treatment with AOAA during the inflammatory peak of immune response can significantly improve DSS-induced colonic injury, which is achieved by balancing macrophage polarization and regulating the secretion of inflammatory cytokines. In addition, the inhibitory effect of AOAA on colitis may be related to the maintenance of TJ and EMT networks. Therefore, our study provides a theoretical basis for the potential application of AOAA in the treatment of IBD.

## Data Availability

The data generated during the present study are available from the corresponding author on reasonable request.

## Supplementary Materials

Supplementary Materials Supplemental table 1 is the DAI scoring criteria of mice. Supplemental table 2 is the quantitative real-time PCR primer sequences.
