# Supplementary material for: Protective Effects of Aminooxyacetic Acid on Colitis Induced in Mice with Dextran Sulfate Sodium
Source: Biomed Res Int. 2021 Dec 10;2021:1477345. doi: 10.1155/2021/1477345 (PMC8923778; doi:10.1155/2021/1477345)
Supplement: Supplementary Materials — Supplemental table 1 is the DAI scoring criteria of mice. Supplemental table 2 is the quantitative real-time PCR primer sequences. [file 1477345.f1.docx]

**Supplementary Materials**

Supplemental Table 1. The DAI scoring criteria of mice.

<0 Normal Negative 0

1-5 + 1

5-10 Loose ++ 2

10-20 +++ 3

>20 Diarrhea Gross bleeding 4

Weight loss (%) Stool consistency Occult blood Score

Five grades of weight loss (0, no loss or weight gain; 1, 1%–5% loss; 2, 5%–10% loss; 3, 10%–20% loss; 4, >20% loss); three grades of stool consistency (0, normal; 2, loose; and 4, diarrhea); five grades of occult blood (0, negative; 1, +; 2, ++; 3, +++; and 4, gross bleeding). DAI values were calculated as the sum of the weight loss score, diarrhea score and rectal bleeding score. The DAI was determined by 3 investigators who were blinded to the experimental conditions.

Supplemental Table 2. The quantitative real-time PCR primer sequences.

| Gene | Forward primer | Reverse primer |
| --- | --- | --- |
| IL-1β | GAAATGCCACCTTTTGACAGTG | TGGATGCTCTCATCAGGACAG |
| TNF- a | CCCTCACACTCAGATCATCTTCT | GCTACGACGTGGGCTACAG |
| IL-6 | CTGCAAGAGACTTCCATCCAG | AGTGGTATAGACAGGTCTGTTGG |
| IL10 | GCTCTTACTGACTGGCATGAG | CGCAGCTCTAGGAGCATGTG |
| CD80 | TGCTGCTGATTCGTCTTTCAC | GAGGAGAGTTGTAACGGCAAG |
| CD206 | CTCTGTTCAGCTATTGGACGC | TGGCACTCCCAAACATAATTTGA |
| Arg1 | CAGAAGAATGGAAGAGTCAG | CAGATATGCAGGGAGTCACC |
| iNOS | CGGACGAGACGGATAGGCAGAG | GGAAGGCAGCGGGCACATG |
| ZO-1 | GCCTGTAAGAGAGGATTCCT | TGTTTCAGGCGAAAGGTAAG |
| Occludin | TGGCGGATATACAGACCCAA | CGATCGTGGCAATAAACACC |
| Claudin1 | CTGTGGATGTCCTGCGTTTC | TCATGCACTTCATGCCAATG |
| E-cadherin | CAGGTCTCCTCATGGCTTTGC | CTTCCGAAAAGAAGGCTGTCC |
| Vimentin | ATGCTTCTCTGGCACGTCTT | AGCCACGCTTTCATACTGCT |
| β-actin | AAATCGTGCGTGACATCAAA | AAGGAAGGCTGGAAAAGAGC |

Sequences of each primer used in the experiments are shown.
